# A novel cytoprotective peptide protects mesenchymal stem cells against mitochondrial dysfunction and apoptosis induced by starvation via Nrf2/Sirt3/FoxO3a pathway

**DOI:** 10.1186/s12967-017-1144-5

**Published:** 2017-02-15

**Authors:** Shuo Wang, Chao Zhang, Sidikejiang Niyazi, Long Zheng, Jiawei Li, Weitao Zhang, Ming Xu, Ruiming Rong, Cheng Yang, Tongyu Zhu

**Affiliations:** 1 Department of Urology, Zhongshan Hospital, Fudan University, 180 Fenglin Road, Shanghai, 200032 China; 2 Shanghai Key Laboratory of Organ Transplantation, Shanghai, China; 3 Shanghai Public Health Clinical Center, Fudan University, Shanghai, China; 4 Department of Transfusion, Zhongshan Hospital, Fudan University, Shanghai, China

**Keywords:** Cyclic helix B peptide, Mesenchymal stem cell, Starvation, Nrf2, Sirt3, FoxO3a

## Abstract

**Background:**

Mesenchymal stem cell (MSC) has been widely explored in the past decade as a cell-based treatment for various diseases. However, poor survival of adaptively transferred MSCs limits their clinical therapeutic potentials, which is largely ascribed to the nutrient starvation. In this study, we determined whether a novel kidney protective peptide CHBP could protect MSCs against starvation and invested the underlying mechanisms.

**Methods:**

MSCs were subjected to serum deprivation and CHBP of graded concentrations was administered. Cell viability and apoptosis were detected by CCK-8, Annexin V/PI assay and Hoechst staining. ROS generation, mitochondrial membrane potential indicated by JC-1 and mitochondrial mass were measured by flow cytometry. The location of cytochrome c within cells was observed under fluorescence microscopy. Expressions of Nrf2, Sirt3, and FoxO3a were analyzed by western blot. In addition, preconditioning MSCs with CHBP was applied to test the possible protection against starvation. Finally, the effect of CHBP on the differentiation and self-renewal capacity of MSCs was also examined.

**Results:**

CHBP improved cell viability and suppressed apoptosis in a dose dependent manner. Starvation resulted in the mitochondrial dysfunction and treatment of CHBP could alleviate mitochondrial stress by diminishing oxidative injury of ROS, restoring mitochondrial membrane potential and maintaining mitochondrial membrane integrity. Importantly, Nrf2/Sirt3/FoxO3a pathway was activated by CHBP and Sirt3 knockdown partially abolished the protection of CHBP. Moreover, MSCs pretreated with CHBP were more resistant to starvation. Under normal condition, CHBP exerted little effects on the differential and self-renewal capacity of MSCs.

**Conclusions:**

The present study demonstrated the efficient protection of CHBP upon MSCs against starvation-induced mitochondrial dysfunction and apoptosis and indicated possible involvement of Nrf2/Sirt3/FoxO3a pathway in the protective effect.

## Background

Mesenchymal stem cell (MSC) is a common adult stem cell originating from the mesoderm and characterized by its multi-potentiality and unique immune-modulatory property [1, 2]. Besides their classic tri-lineage differentiation into adipocytes, osteoblasts, and chondrocytes, it is reported that MSCs can also differentiate into ectodermal and endodermal cells, such as endothelial cells, smooth muscle cells, hepatocytes, and cardiomyocytes [3, 4]. Importantly, as adult stem cells, MSCs can be easily isolated and feasibly expanded ex vivo [5]. In this respect, MSC is considered as an ideal cell-based therapy.

An increasing body of evidence has demonstrated that MSCs hold great promise for the therapy of tissue injuries, inflammatory diseases, and allograft rejections [6–12]. However, in most cases the survival rate of adoptively transferred MSCs is poor, which significantly hampers the treatment efficiency [13–15]. Although the therapeutic capability of MSCs, in the settings of some diseases, might rely on regulating inflammation and enhancing endogenous tissue repair [16, 17], longer survival and retention time would optimize MSC-based therapy efficacies. The reason that transplanted MSCs are short-lived could be largely ascribed to the complex microenvironments. After adoptive transfer, MSCs will encounter with various undesirable factors including nutrient deprivation, oxidative stress, inflammation reactions and so on, all of which could decrease the cell viability and thereby compromise their therapeutic activities [18–21]. Nutrient starvation is one of the major obstacles confronted by engrafted MSCs, especially within injured tissues. In spite that appropriate preconditioning with hypoxia and serum starvation could augment the cellular viability, prolonged exposure to starvation will be a lethal disaster for MSCs [22, 23]. Therefore, improving survival of MSCs under starvation conditions is of vital importance for the MSC-based cell therapies.

Cyclic helix B peptide (CHBP) is a novel erythropoietin-derived peptide synthesized by our group recently with prominent renoprotective effect [24–27]. Our previous studies have demonstrated that CHBP could alleviate kidney ischemia–reperfusion injury through inhibiting apoptosis as well as inflammatory responses [25, 26]. However, whether CHBP is able to exert cytoprotection in the background of starvation remains elusive. In the present study, we adopted the serum deprivation strategy to mimic the starvation during MSCs’ administration. Treatment of CHBP significantly improved mitochondrial dysfunction, prevented apoptosis and prolonged cell survival. Moreover, we revealed that CHBP exerted its cytoprotection through Nrf2/Sirt3/FoxO3a pathway. This research provided a novel pharmacological recognition of CHBP for the cytoprotective effects and suggested CHBP as a promising adjuvant agent in cell-based treatments.

## Methods

### Cell culture and treatment

Primary bone marrow-derived MSCs of C57/BL6 mice were purchased from Cyagen Biosciences Inc. (Guangzhou, China). MSCs were cultured in Dulbecco’s modified Eagle’s medium/F12 (DMEM/F12) medium with 10% heat-inactivated fetal bovine serum, 2 mmol/L glutamine, 100 U/ml penicillin, and 100 mg/ml streptomycin at 37 °C in a humidified incubator containing 5% CO_2_. All experiments were performed on MSCs from 6th to 10th passages. Apoptosis was induced by serum deprivation. Briefly, after MSCs were seeded into 6-well plates or 96-well plates, the culture medium was replaced with serum-free DMEM low glucose with or without CHBP at various concentrations (0.1, 1, 10 nmol/L). MSCs maintained in complete medium were used as the normal control. To exclude a nonspecific impact of peptide itself, a scrambled control peptide (LSEARNQSEL) was also used. Both CHBP and scrambled peptide were dissolved in PBS.

### Cell viability

Cell viability in response to starvation was examined by Cell Counting Kit-8 (CCK-8, Dojindo, Shanghai, China). Cells were seeded in 96-well plates at a density of 1 × 10^4^ cells/well. After different treatment, 10ul of CCK-8 reagent was added to each well of the 96-well plates and incubated at 37 °C for 2 h. The absorbance of each sample at 450 nm was measured by a microplate reader.

Meanwhile, trypan blue staining was used to distinguish cell death. MSCs were seeded in 6-well plates 1 × 10^6^ cells/well cells and treated differently. Then non-adherent and adherent cells were harvested and resuspended in 1 ml complete medium. 5 μl of these cell suspensions were aspirated and mixed with an equal volume of 0.4% trypan blue solution, and finally counted using a hemacytometer under microscope.

### Annexin V/PI assay

To further explore the protection of CHBP against starvation, cell death was analyzed using Annexin V–FITC/PI apoptosis detection kit (Vazyme Biotech, Nanjing, China). According to the manufacturer’s protocol, cells were harvested and resuspended in 100 μl binding buffer mixed with 5 μl Annexin V-FITC reagent and 5 μl PI reagent. After incubation for 15 min at room temperature in the dark, another 400 μl binding buffer was added, and cells were measured by flow cytometry (Beckman Coulter). Data was analyzed with FlowJo software.

### Hoechst staining

Hoechst 33258 (Beyotime, Nanjing, China) was used for to examine the morphological changes of apoptosis. MSCs with different treatment were fixed in 4% paraformaldehyde for 2 h and subsequently stained with Hoechst 33258 reagent for 30 min at room temperature. Nuclear alterations were then observed under fluorescence microscopy. Fragmentation and condensation of the nucleus were recognized as the characteristics of apoptotic cells.

### Reactive oxygen species (ROS), mitochondrial membrane potential (MMP), and mitochondrial mass detection

Briefly, for quantitative detection of mitochondria-derived ROS, MMP and mitochondrial mass, cells were incubated with MitoSOX reagent (2.5 mmol/L, Invitrogen), JC-1 reagent (5 mg/mL, Beyotime), and MitoRed (50 nmol/L, KeyGEN Biotech) for 30 min at 37 °C. Subsequently, MSCs were collected, washed with PBS and then analyzed by flow cytometry.

### Immunofluorescence staining

Cells were first incubated with 50 nmol/L MitoRed for 30 min at 37 °C. To examine cytochrome c location, cells were fixed in 4% paraformaldehyde for 2 h, permeabilized with 0.2% Triton X-100 for 5 min. After blocked with 10% bovine serum albumin for 1 h at room temperature, cells were incubated with primary antibody against cytochrome c (1:500, Beyotime) overnight at 4 °C, and subsequently incubated with FITC-conjugated secondary antibodies (1:1000, Beyotime). Locations of mitochondria and cytochrome c were visualized under fluorescence microscopy.

### Knockdown of Sirt3 by small interfering RNA (siRNA)

For knockdown of Sirt3 in vitro, siRNA targeting Sirt3 (ThermoFisher Scientific, AM16708) was used. The siRNA was transfected into MSCs using Lipofectamine 3000 (Invitrogen) according the manufacturer’s instructions. Briefly, MSCs were seeded in 6-well plates with serum-free DMEM medium, and then subjected to the mixture of siRNA and Lipofectamine 3000 reagent. After incubation for 6 h, medium was changed and the cells were harvested for the further experiments.

### Western blotting analysis

Briefly, total proteins from MSCs were separated on SDS–polyacrylamide gels, transferred onto nitrocellulose membranes, blocked and incubated with anti-Nrf2, anti-Sirt3, anti-total FoxO3a, and phosphorylated-FoxO3a (p-FoxO3a, Ser253 and Ser318/321) antibodies (1:1000, Cell Signaling Technology) and anti-tubulin antibodies (1:10000, Abcam) overnight at 4 °C. Membranes were then washed 3 times and incubated with secondary antibodies for 1 h at room temperature. The semi-quantitative analysis (AlphaView Software 3.3, Cell Biosciences, Inc.) results were expressed as the optical volume densities (OD × mm^2^) normalized to tubulin.

### MSC differentiation

For osteogenic differentiation, MSCs were cultured in osteogenic differentiation medium containing 10% FBS, 0.2 mmol/L ascorbate, 10 mmol/L b-glycerolphosphate, 0.1 mmol/L dexamethasone, 100 U/ml penicillin and 100 mg/ml streptomycin (all from Cyagen, Guangzhou, China) at the confluence of 60–70%. Osteogenic medium was changed every 3 days. After induction for 21 days, cells were fixed by 4% formaldehyde, and then stained with Alizarin Red S to assess calcium deposits.

MSCs at 100% confluence were used for adipogenic differentiation. Cells were first cultured in adipogenic induction medium supplemented with 10% FBS, 1 mmol/L dexamethasone, 100 mmol/L indomethacin, 0.5 mmol/L methyisobutylxanthine, 10 mmol/L insulin, 100 U/ml penicillin and 100 mg/ml streptomycin for 3 days, followed by cultivating in maintenance medium consisting of 10% FBS, 5 mg/ml insulin, 100 U/ml penicillin and 100 mg/ml streptomycin for 24 h. After 4 cycles of induction/maintenance exchange, MSCs were cultured in maintenance medium for 6 days and finally fixed by 4% formaldehyde. Lipid droplets were examined using oil red O solution staining.

### Colony forming unit (CFU) assay

To test the self-renewal capacity of CHBP-treated MSCs, CFU assay was conducted. In brief, MSCs were plated in 10 cm dishes at a density of 50 cells/cm^2^ and cultured in normal complete medium in the presence or absence of 10 nM CHBP for 14 days, with medium exchange every 3 days. At the end of cultivation, cells were washed with PBS, fixed by 4% formaldehyde for 30 min and stained with 2% crystal violet for 10 min. Subsequently, cells were washed with PBS for 3 times and left drying. Colonies consisting of more than 50 cells were defined as CFUs and were counted.

### Statistical analysis

All data are presented as mean ± standard deviation. Statistical analysis was performed using the Student’s *t* test or one-way ANOVA by SPSS 19.0 software (SPSS Inc., Armonk, NY, USA). *P* < 0.05 was recognized as statistically significant.

## Results

### CHBP preserved the viability of MSCs during starvation

**Fig. 1:**
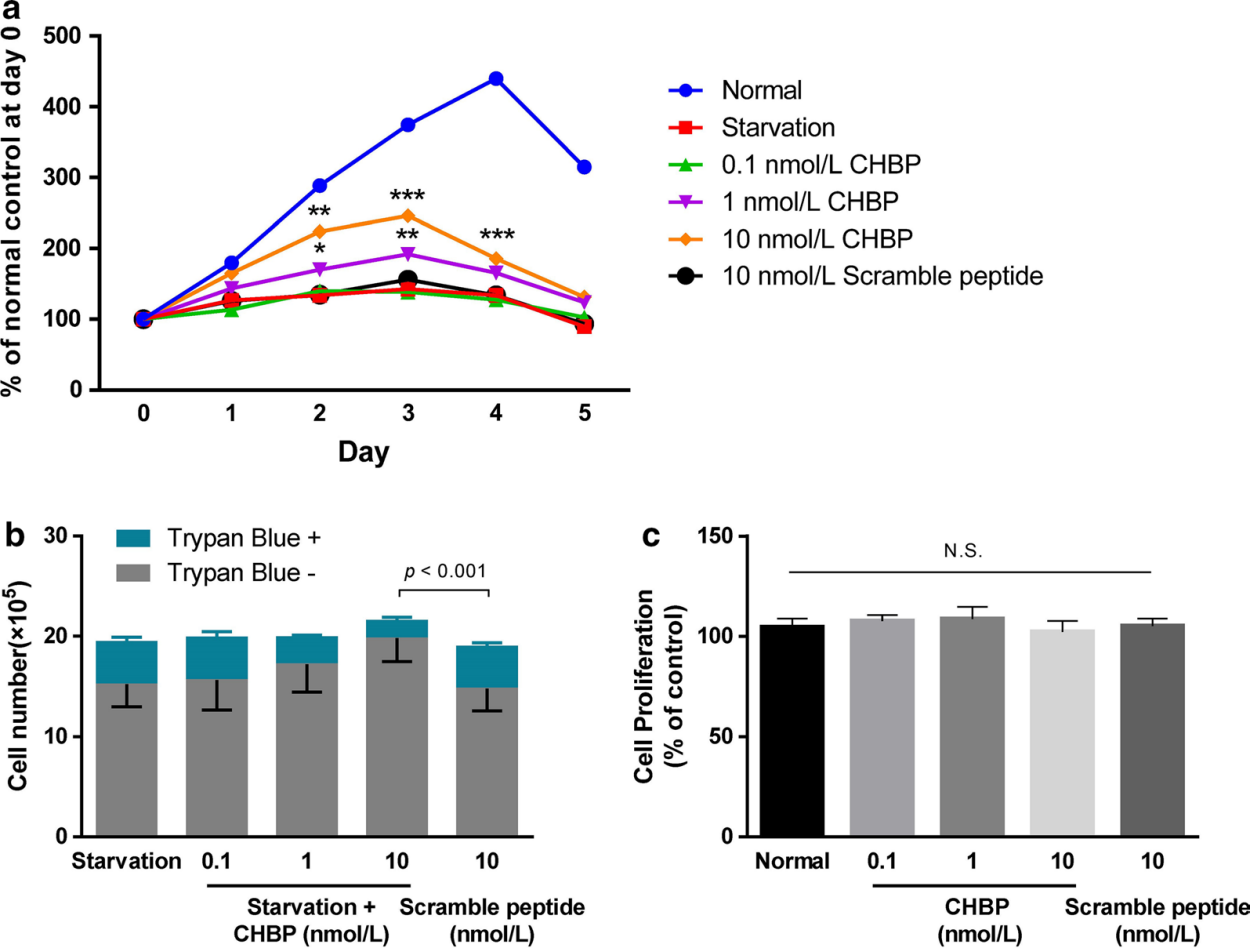
CHBP preserved the viability of MSCs during starvation. **a** MSCs were seeded in 96-well plates at a density of 1 × 10^4^ cells/well and subjected to starvation. CHBP was administered at indicated concentrations. Scrambled peptide was also used as a control. Cell viability was determined by CCK-8. **b** MSCs seeded in 6-well plates at 1 × 10^6^ cells/well were incubated in serum-free medium for 24 h in the absence or presence of CHBP and harvested for Trypan blue staining. **c** Effect of CHBP on the proliferation of MSCs under normal conditions was measured by CCK-8. Experiments were performed in triplicate. Data are expressed as mean ± standard deviation. *P < 0.05, **P < 0.01, ***P < 0.001

To examine the possible protection of CHBP, cell viability of nutrient-starved MSCs treated with different concentrations of CHBP (0, 0.1, 1, 10 nmol/L) was analyzed using CCK-8 assay. Figure 1a exhibited the dynamics of cell viability during starvation. Serum deprivation, just as expected, caused significant decrease of cell viability compared with normal control. CHBP were able to partially alleviate the decrease of viability in a dose-dependent manner, and the protective effect was observed for up to 72 h of starvation. We observed that starvation resulted in the detachment of cells, which usually represented cell death. Therefore, we further used Trypan blue staining to determine loss of cell membrane integrity and found that CHBP could obviously inhibit starvation-induced cell death (Fig. 1b). To exclude a possible protective effect of peptide molecule itself, a scrambled peptide was used and identified no detectable protection. To examine whether CHBP itself has an effect on the proliferation of MSCs, we subjected MSCs to CHBP at various concentrations in complete medium. As shown in Fig. 1c, CHBP had no detectable impact on the proliferation of MSCs at the concentration used in the present study. Collectively, our data suggested that CHBP could preserve cell viability against starvation by alleviating cell death.

### CHBP attenuated starvation-induced apoptosis in MSCs

**Fig. 2:**
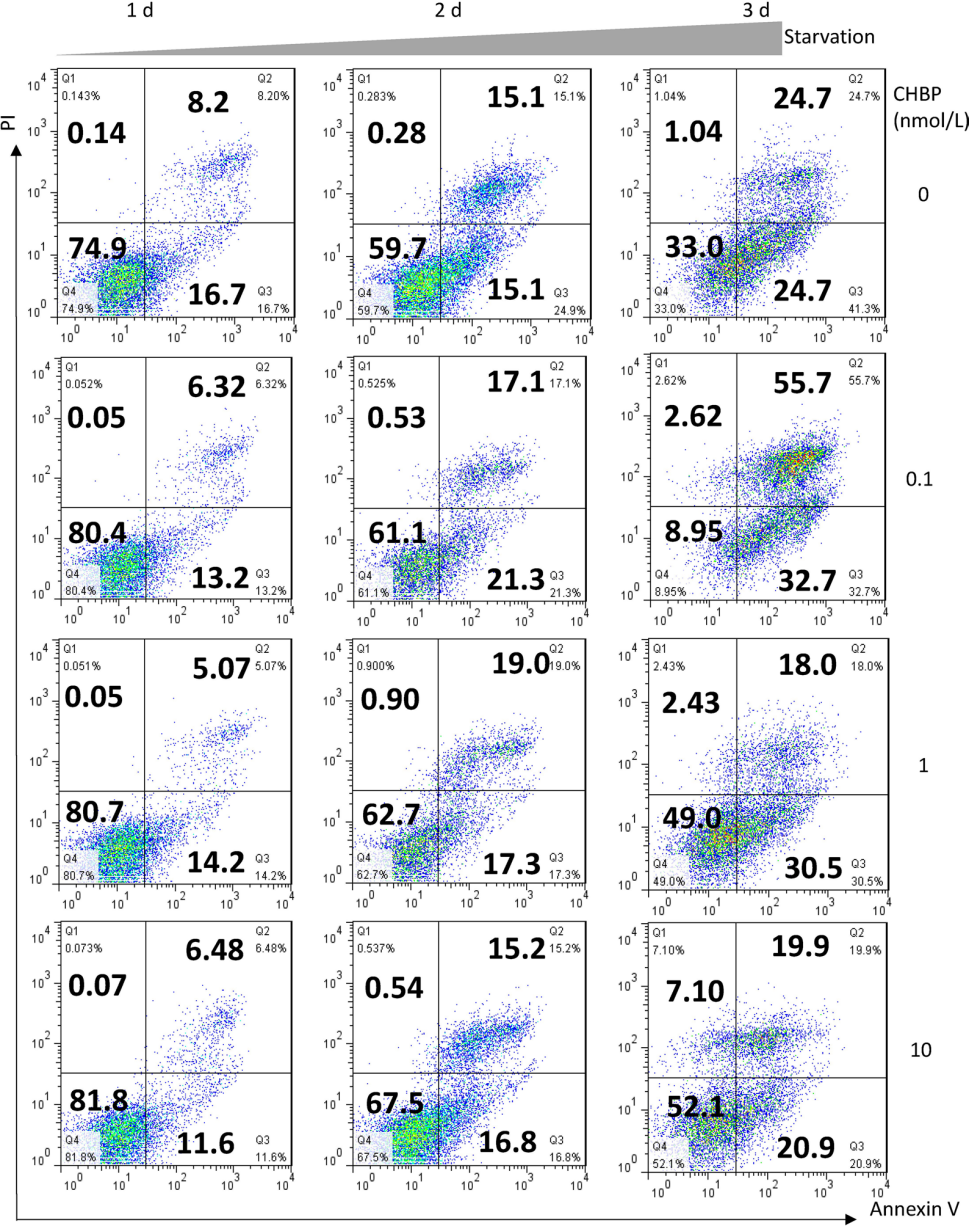
CHBP suppressed apoptosis induced by serum deprivation. MSCs were seeded in 6-well plates at a concentration of 1 × 10^6^/well and incubated in serum-free medium for indicated time. The protective effect of CHBP against apoptosis was evaluated by Annexin V/PI asaay and Hoechst 33258 staining. **a** Annexin V/PI asaay was performed by flow cytometry. Annexin V−/PI− represents live cells; Annexin V+/PI− or Annexin V−/PI+ represents early or late apoptosis phase respectively; Annexin V+/PI+ reflects necrosis. Fragmentation and condensation of the nucleus were recognized as the characteristics of apoptotic cells. Experiments were performed in triplicate. Data are expressed as mean ± standard deviation. *P < 0.05 compared with control. **P < 0.01 compared with control

**Fig. 3:**
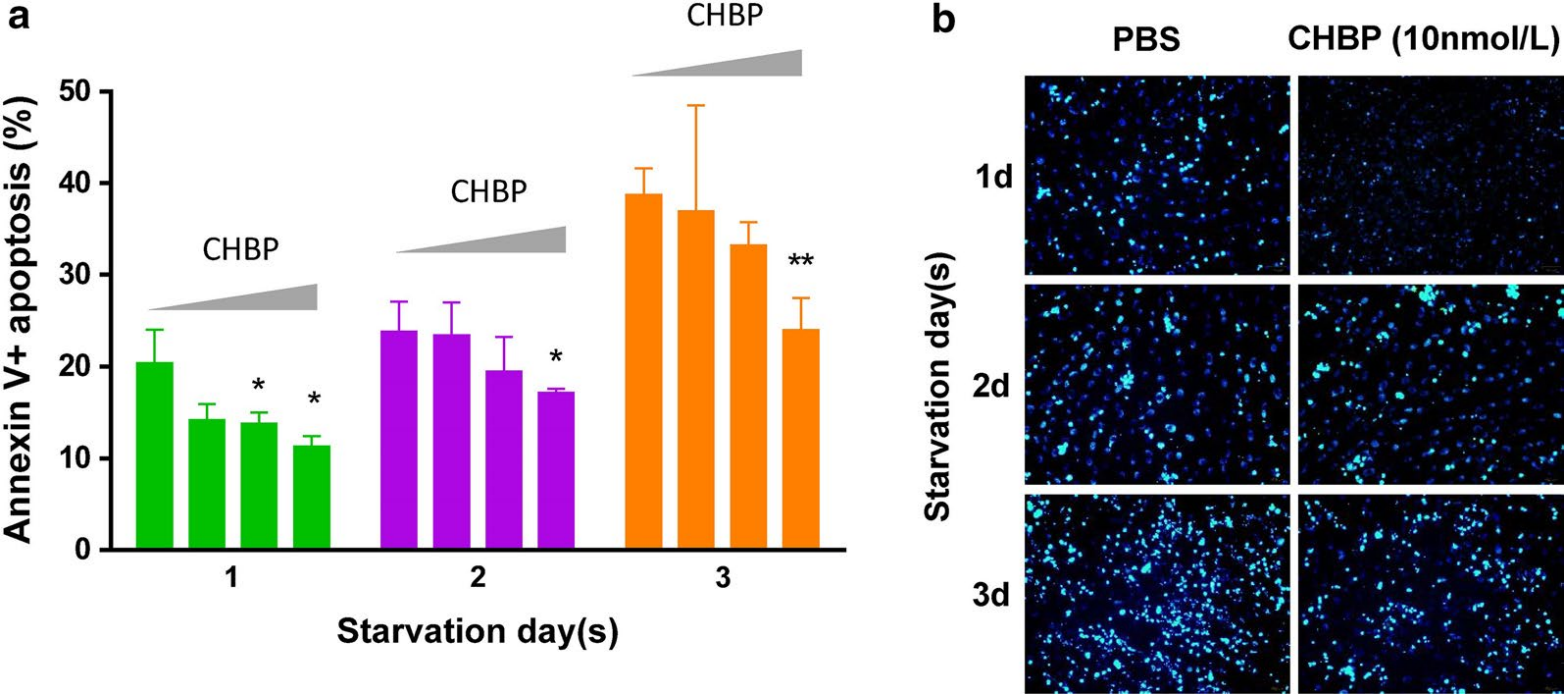
CHBP suppressed apoptosis induced by serum deprivation. MSCs were seeded in 6-well plates at a concentration of 1 × 10^6^/well and incubated in serum-free medium for indicated time. The protective effect of CHBP against apoptosis was evaluated by Annexin V/PI asaay and Hoechst 33258 staining. **a** Quantification analysis of flow cytometry results was performed and demonstrated. **b** Hoechst 33258 staining was observed under fluorescence microscopy at ×100 magnification. Fragmentation and condensation of the nucleus were recognized as the characteristics of apoptotic cells. Experiments were performed in triplicate. Data are expressed as mean ± standard deviation. *P < 0.05 compared with control. **P < 0.01 compared with control

To further delineate the effect of CHBP in starvation-induced cell death, apoptosis and necrosis was qualitatively analyzed using Annexin V/PI assay by flow cytometry. Annexin V−/PI− represents live cells; Annexin V+/PI− or Annexin V−/PI+ represents early or late apoptosis phase respectively; Annexin V+/PI+ reflects necrosis. As suggested in Figs. 2a, 3a, starvation induced significant apoptosis (Annexin V+) that exacerbated over time and CHBP, in contrast, markedly inhibited apoptosis in a concentration dependent manner. The highest concentration (10 nmol/L) was therefore applied for the following experiments. The protective effect of CHBP was also confirmed using Hoechst staining. Nuclear condensation observed in starved MSCs was obviously prevented by CHBP treatment (Fig. 3b). Taken together, these results indicated that CHBP attenuated starvation-induced apoptosis in MSCs.

### CHBP improved mitochondrial dysfunction in response to serum deprivation

**Fig. 4:**
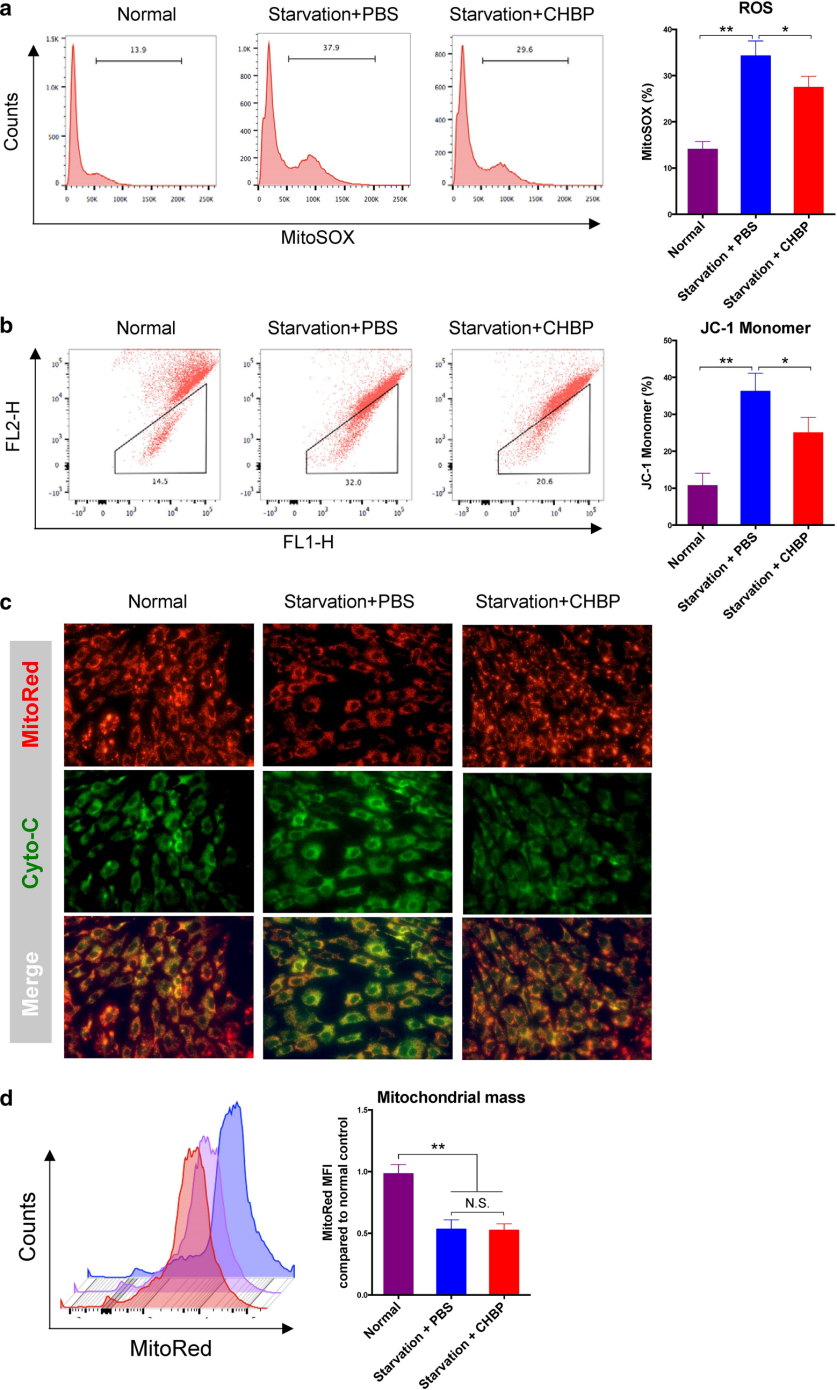
CHBP prevented mitochondrial dysfunction. MSCs were seeded in 6-well plates at a concentration of 1 × 10^6^/well and incubated in serum-free medium for 24 h with or without 10 nmol/L CHBP. MSCs cultured in complete medium were used as control. **a** To detect of mitochondria-derived ROS, cells were incubated with MitoSOX reagent (2.5 mmol/L) for 30 min and analyzed by flow cytometry. **b** To measure mitochondrial membrane potential, cells were incubated with JC-1 reagent (5 mg/mL) for 30 min and analyzed by flow cytometry. **c** Cytochrome c was labeled by FITC and mitochondria were stained by MitoRed (50 nmol/L). Cells were observed by fluorescence microscopy under ×200 magnification. **d** For detection of mitochondrial mass, cells were incubated with MitoRed (50 nmol/L) for 30 min and analyzed by flow cytometry. Experiments were performed in triplicate. Data are expressed as mean ± standard deviation. *P < 0.05, **P < 0.01

Mitochondria are the main energy-producing organelles within cells and also play a key role in regulating cell bioactivity. Previous studies have shown that serum deprivation could result in mitochondrial dysfunction, which acted as an important contributor to apoptosis [28–30]. Therefore, we explored whether CHBP were able to diminish mitochondrial stress. Mitochondria are the main source of intracellular ROS, which is an inevitable product of the respiratory chain during oxidative phosphorylation. Excessive ROS accumulate during pathological conditions and cause oxidative damage. Several studies indicated that ROS might serve as an initiator of the mitochondrial change [31, 32]. As shown in Fig. 4a, serum deprivation led to an obvious increase of ROS, which was significantly alleviated by CHBP. MMP is recognized as a marker of mitochondrial statement. Physiologically, JC-1 is prone to form aggregates that are fluorescent red depending on the polarization of MMP; in contrast, when MMP collapses, JC-1 disperses into monomers that are fluorescent green. Our data suggested that CHBP could help maintain a normal polarized MMP (Fig. 4b). Mitochondrial injury damages the integrity of mitochondrial membrane and leads to the leakage of cytochrome c, a promoter of inner apoptosis pathway. Normally, as shown in Fig. 4c, cytochrome c labeled with FITC colocalized with MitoRed-stained mitochondria. Under starvation, green fluorescence dramatically increased, indicating a release of cytochrome c from mitochondria. Consistent with the ROS generation and MMP results, CHBP inhibited mitochondrial membrane breakdown. However, CHBP did not prevented the decrease in starvation-associated mitochondrial mass (Fig. 4d).

### Nrf2/Sirt3/FoxO3a pathway may involve in the protection of CHBP against serum deprivation

**Fig. 5:**
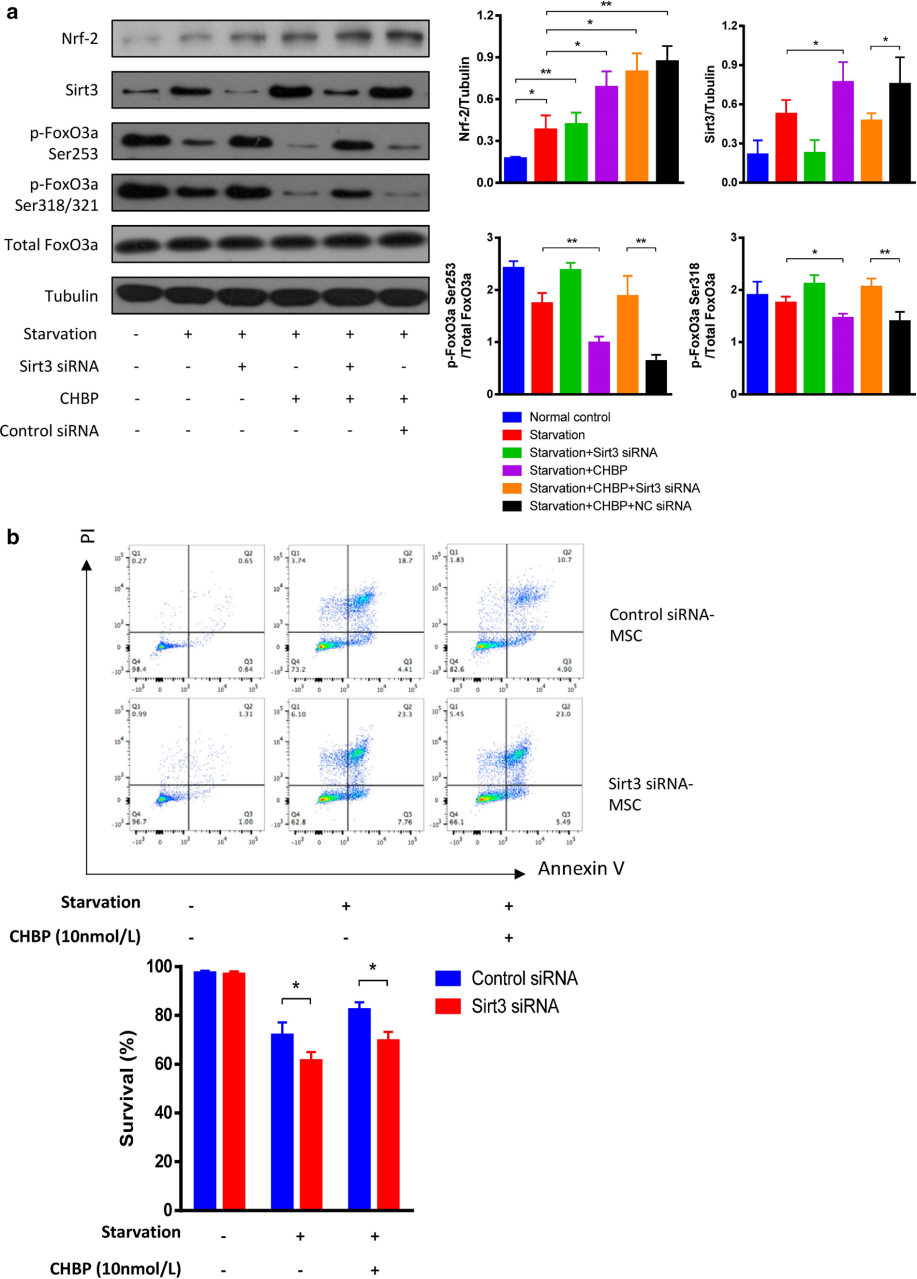
Nrf2/Sirt3/FoxO3a pathway participated in the protective mechanism of CHBP against starvation. **a** MSCs were treated with siRNA targeting Sirt3 and then subjected to starvation with CHBP (10 nmol/L). Total protein was prepared from cultured cells. Expressions of Nrf2, Sirt3, total FoxO3a and p-FoxO3a were analyzed by western blotting. **b** Sirt3 siRNA treated MSCs were subjected to serum deprivation and apoptosis was detected by Annexin V/PI asaay. Experiments were performed in triplicate. Data are expressed as mean ± standard deviation. *P < 0.05, **P < 0.01

Previous studies have shown that erythropoietin could upregulate nuclear factor erythroid 2-related factor 2 (Nrf2), which could further induce Sirt3 expression to exert protective effect. Therefore, we presumed that CHBP, as an erythropoietin deviant, might protect MSCs against starvation stress via Nrf2/Sirt3 related pathway. To confirm this hypothesis, we detect the expression of Nrf2, Sirt3, total FoxO3a, and p-FoxO3a by western blot. As shown in Fig. 5a, Nrf2 and Sirt3 were significantly upregulated by CHBP, while p-FoxO3a was suppressed. It is well accepted that FoxO3a plays a crucial role in mitochondrial protection and the phosphorylation of FoxO3a can prevent its activation [33]. To further determine the role of Sirt3/FoxO3a pathway in the effect of CHBP, Sirt3 siRNA was used. Compared with negative control (NC) siRNA-treated group, Sirt3 siRNA increased the level of p-FoxO3a and thereby inhibited the activation of FoxO3a. The NC siRNA were prone to downregulate p-FoxO3a, which, however, did not reach the statistic significance (Fig. 5a). Furthermore, we repeated the apoptosis assay by flow cytometry using Sirt3 siRNA-treated MSCs. As indicated in Fig. 5b, Sirt3 knockdown partially abolished the protection of CHBP, indicating a certain role of sirt3 in the protective effect of CHBP. Taken together, all these results suggested that Nrf2/Sirt3/FoxO3a pathway might participate in the protection of CHBP on MSCs against serum deprivation.

### MSCs pretreated with CHBP were more resistant to starvation

**Fig. 6:**
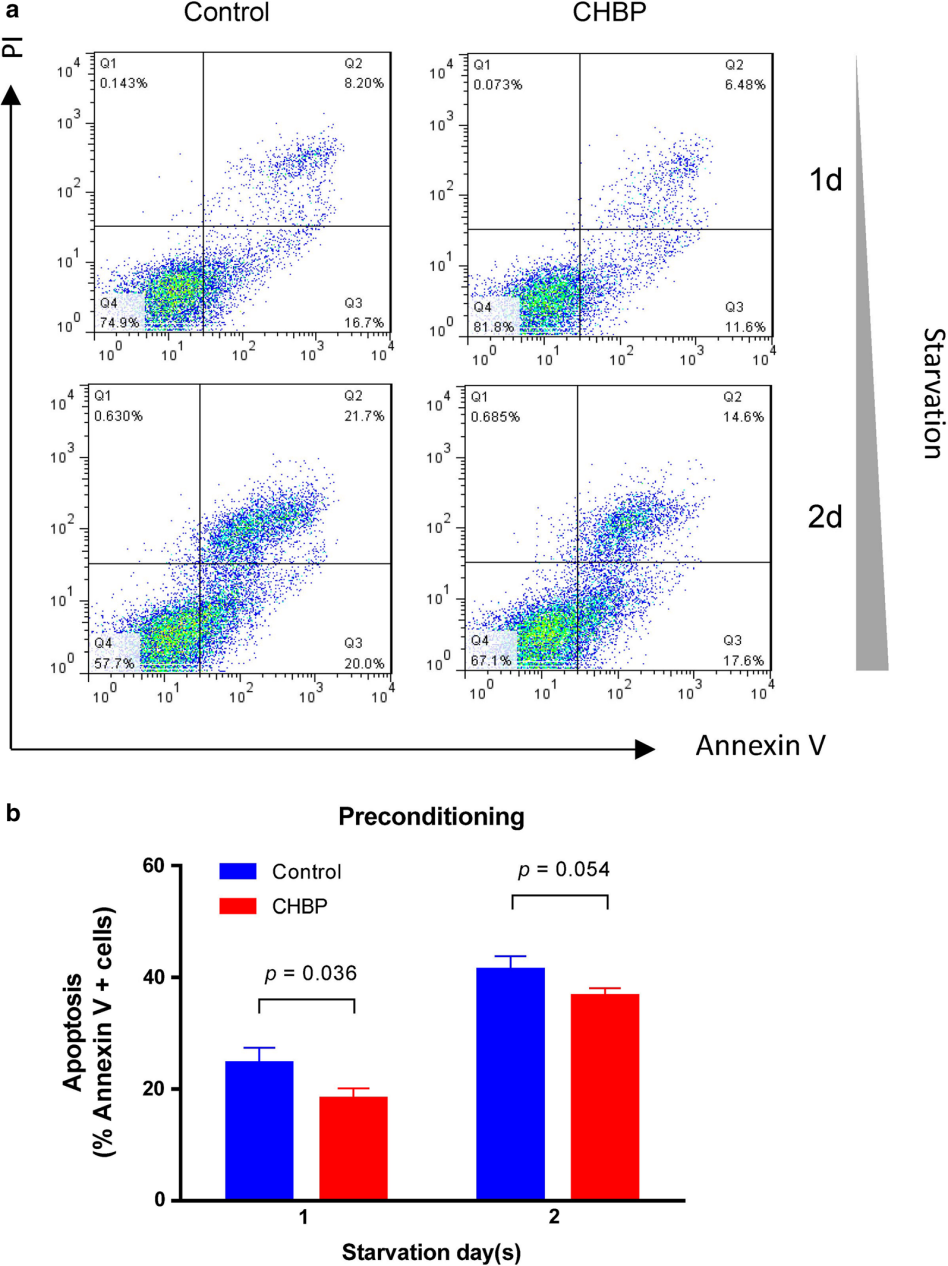
CHBP preconditioning rendered MSCs more resistant to starvation. **a** MSCs were preconditioned with CHBP at a concentration of 10 nmol/L and then subjected to serum starvation. Apoptosis was detected by Annexin V/PI asaay. **b** Quantification analysis of flow cytometry results was performed and demonstrated. Experiments were performed in triplicate. Data are expressed as mean ± standard deviation

Pharmacological preconditioning is a well-accepted strategy to improve poor survival of transplanted cells. To investigate the potential protection of CHBP in a pretreatment manner, MSCs were pretreated with CHBP for 24 h and then subjected to serum-free medium. The results demonstrated that preconditioning of CHBP could also significantly inhibit apoptotic cell death in MSCs during serum deprivation (Fig. 6a, b). The protective effect was observed for up to 48 h of starvation, although the decrease of apoptosis at 48 h did not reach a statistical significance (*P* = 0.054). However, preconditioning MSCs with CHBP for 24 h failed to improve the viability at 72 h (data not shown).

### CHBP exerted little effects on the differential and self-renewal capacity of MSCs

**Fig. 7:**
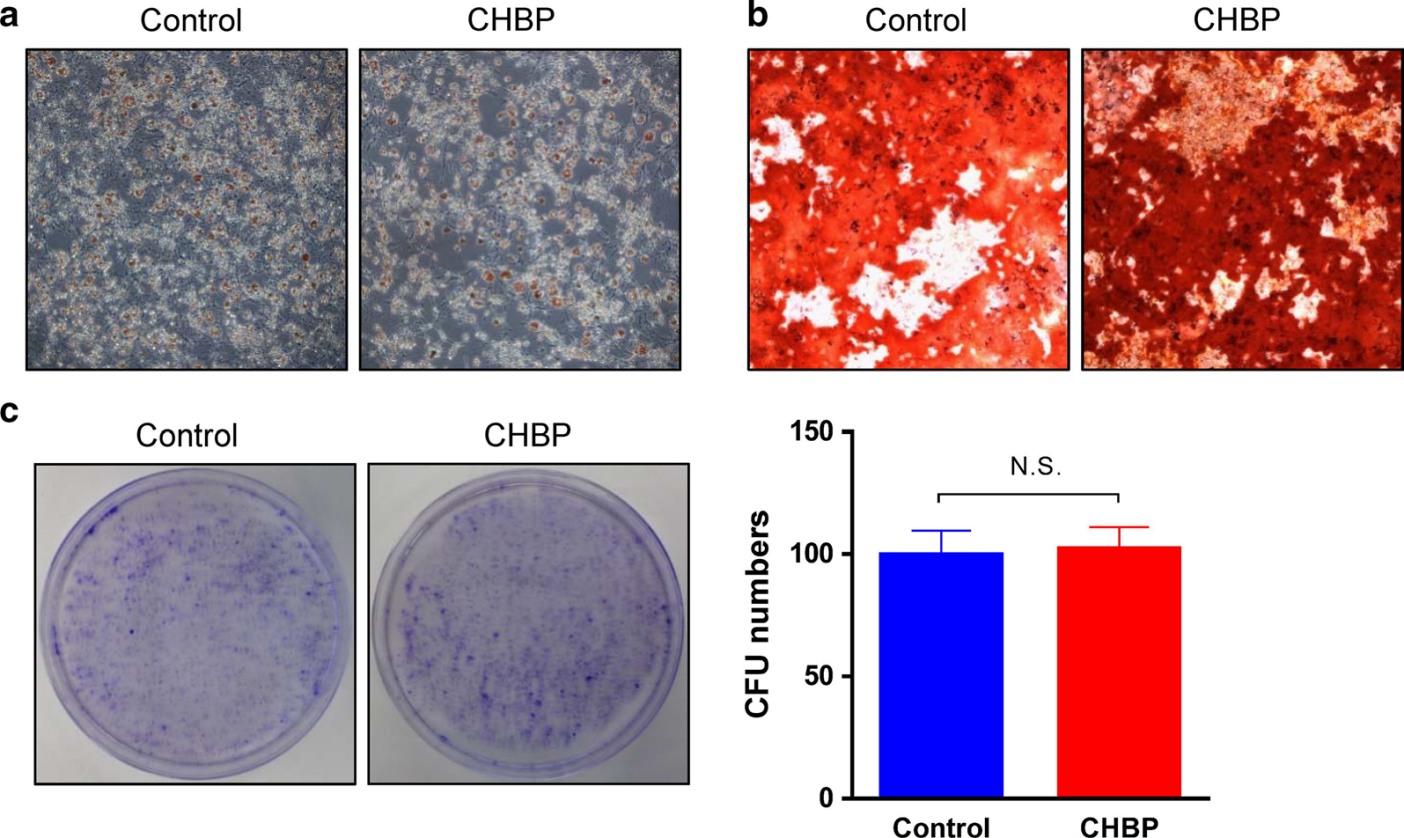
CHBP had no impact on the differential and self-renewal capacity of MSCs. MSCs were incubated with CHBP at a concentration of 10 nmol/L for 3 days and then harvested for **a** adipogenic differentiation, **b** osteogenic differentiation, and **c** Colony forming unit assay. Photographs are representatives of three independent experiments. Data are expressed as mean ± standard deviation

Multipotential differentiation and self-renewal capacities are hallmarks to define a stem cell. MSCs, as adult stem cells, are characterized by their differentiations into osteoblasts and adipocytes. To test whether treatment of CHBP could affect these biological activities, differentiation and CFU assay were performed. As shown in Fig. 7a, b, preconditioning MSCs with CHBP at 10 nmol/L in normal medium for 3 days has no significant impact on the differential and self-renewal capability.

## Discussion

In the past decade, there has been considerable interest in the exploration of MSCs as cell-based treatment for various diseases. However, poor survival of adaptively transferred MSCs limits their clinical therapeutic potential, which to a great extent is due to the nutrient starvation. Therefore, it is of vital importance to discover regimens that improve the viability of starved MSCs. This study aims to determine whether the organ-protective peptide CHBP can rescue MSCs that were subjected to serum deprivation. Serum deprivation is a widely used model of starvation that could initiate the intrinsic apoptotic pathway [34]. In the present study, we confirmed that serum deprivation could result in apoptosis and exacerbate cell death as starvation time increased. It is demonstrated that CHBP could limit apoptosis of MSCs in a concentration-dependent manner. To detect whether this protection is a by-product effect of peptide molecule itself, a scrambled peptide is used as a control. No detectable protection was recognized for the scrambled peptide. Although erythropoietin and its deviants have been shown cytoprotection in the background of starvation, the underlying mechanisms are not fully elucidated [35–38].

Mitochondria, in addition to the well-known energy-producing function, also serve as a crucial mediator of cell injury and death [39, 40]. Physiologically, the homeostasis of mitochondria is finely orchestrated by a complex network consisting of intracellular proteins and microenvironments. Upon severe stresses, such homeostasis can be dramatically damaged, leading to mitochondrial dysfunction and consequent cell death. Consistent with previous studies [28–30, 41], out data showed that serum deprivation induced mitochondrial dysfunction, resulting in increased ROS, impaired MMP and release of cytochrome c. ROS is an inevitable product of the respiratory chain during oxidative phosphorylation. Pathological stimuli such as nutrient starvation cause a functional disruption of mitochondria and consequently result in excessive ROS generation, which induced undesirable oxidative damage to DNA, proteins, and organelles and even lead to cell death [42–44]. Simultaneously, as the main source of ROS, mitochondria itself acts as a preferable target for the deleterious effect of ROS. Oxidative stress in turn deteriorates mitochondrial dysfunction and amplifies mitochondrial collapse in a positive feedback loop. In contrast, CHBP could alleviate mitochondrial stress by diminishing oxidative injury of ROS, restoring MMP and maintaining mitochondrial membrane integrity.

Our results further indicated that antioxidant Nrf2/Sirt3/FoxO3a axis might involve in the protection of CHBP against serum deprivation. Nrf2, also known as GA-binding protein, is a transcription factor belonging to E26 transformation-specific factor family [45]. Nrf2 interacts with PGC-1α to regulate key functional signaling within mitochondria and actives the expression of many cytoprotective proteins including Sirt3 [46–48]. Previous studies have shown that erythropoietin could induce Nrf2 expression [49–51]. Accordingly, we found that CHBP was also able to upregulate Nrf2 as well as Sirt3 in the background of starvation. Very recently, increasing evidence demonstrated that Sirt3/FoxO3a pathway served as an important contributor to the mitochondrial protection in response to cellular stresses [52–54]. Sirt3 is a mitochondrial deacetylase involving in several important metabolic processes [55, 56]. The Sirt3-mediated deacetylation of FoxO3a further reduces FoxO3a phosphorylation [53]. The function of FoxO3a is critically modulated by phosphorylation: phosphorylated FoxO3a retains in the cytoplasm and keeps functionally inactive; in contrast, dephosphorylation leads to its transfer to the nucleus to activate target genes [33]. Our results provided direct evidence for the activation of FoxO3a by Sirt3 and revealed that Sirt3 knockdown partially abolished the protection of CHBP.

Scientific community has paid much attention to improve the viability of MSCs in response to nutrient deprivation. Treatment of various reagents is considered as a feasible procedure to protect MSCs from starvation. Among them, there are hormone, endogenous substance, clinically approved drug, peptide and energy resource ATP [57–62]. Neuropeptide substance P (SP) is a peptide containing 11 amino acids and has been suggested to inhibit starvation-induced apoptosis by enhancing Wnt pathway [60]. Moreover, another study shows that the glucose-dependent insulinotropic peptide (GIP) exerts an anti-apoptotic function by regulating adenylate cyclase [58]. These studies suggested that peptides serve as effective adjuvant agents for improving MSC-based therapies. As a peptide, CHBP is relatively low-cost, feasible for large-scale production and stable under various pathophysiologic conditions. In addition, no obvious effect of CHBP is observed upon the osteogenic and adipogenic differentiation, as well as the self-renewal capacity that are crucial hallmarks of MSCs. In this regard, CHBP shows a promising prospect in the clinical practice.

## Conclusions

In conclusion, the present study demonstrates the efficient protection of CHBP upon MSCs against starvation-induced apoptosis and unveils the underlying mechanism of this protective effect. These results provide a better understanding of the multiple pharmacologic functions of CHBP and indicate a possible role of CHBP in regulating nutrient-associated biological processes. Our study suggests that CHBP holds great promise for sustaining cell survival under nutrient-deprived conditions and improving the therapeutic effectiveness of MSC-based treatment. However, the efficacy of CHBP-treated MSCs needs further confirmation in vivo in the future.

## Abbreviations

CHBP: cyclic helix B peptide
DMEM: Dulbecco’s modified Eagle’s medium
MMP: mitochondrial membrane potential
MSC: mesenchymal stem cell
ROS: reactive oxygen species
Nrf2: nuclear factor erythroid 2-related factor 2
siRNA: small interfering RNA
